# Study of Atomic Layer Deposition Nano-Oxide Films on Corrosion Protection of Al-SiC Composites

**DOI:** 10.3390/ma16186149

**Published:** 2023-09-10

**Authors:** Hou-Jen Chen, Ying-Chu Chen, Pi-Chen Lin, Kaifan Lin, Jonathan C. Lin, Miin-Jang Chen, Hsin-Chih Lin

**Affiliations:** 1 Department of Materials Science and Engineering, National Taiwan University, Taipei 106319, Taiwan; f10527a06@ntu.edu.tw (H.-J.C.); r09527018@ntu.edu.tw (Y.-C.C.); royroy162000@gmail.com (P.-C.L.); kafainlin@gmail.com (K.L.); mjchen@ntu.edu.tw (M.-J.C.); 2 Huang Chieh Metal Composite Material Technology Company, Limited, New Taipei 238007, Taiwan; j-lin@hk-metal.cn

**Keywords:** atomic layer deposition, aluminum matrix composite, corrosion protection

## Abstract

In recent years, aluminum matrix composites (AMCs) have attracted attention due to their promising properties. However, the presence of ceramic particles in the aluminum matrix renders AMCs a high corrosion rate and makes it challenging to use traditional corrosion protection methods. In this study, atomic layer deposition (ALD) techniques were used to deposit HfO_2_, ZrO_2_, TiO_2_, and Al_2_O_3_ thin films on AMC reinforced with 20 vol.% SiC particles. Our results indicate that the presence of micro-cracks between the Al matrix and SiC particles leads to severe micro-crack-induced corrosion in Al-SiC composites. The ALD-deposited films effectively enhance the corrosion resistance of these composites by mitigating this micro-crack-induced corrosion. Among these four atomic-layer deposited films, the HfO_2_ film exhibits the most effective reduction in the corrosion current density of Al-SiC composites in a 1.5 wt% NaCl solution from 1.27 × 10^−6^ A/cm^2^ to 5.89 × 10^−11^ A/cm^2^. The electrochemical impedance spectroscopy (EIS) investigation shows that HfO_2_ deposited on Al-SiC composites has the largest R_p_ value of 2.0 × 10^16^. The HfO_2_ film on Al-SiC composites also exhibits effective inhibition of pitting corrosion, remaining at grade 10 even after 96 h of a salt spray test.

## 1. Introduction

In order to harness the characteristics of multiple materials, various composite materials have been developed to utilize the complementary effects between these materials. Among these composites, metal matrix composites take metals as the main components and add ceramic particles to enhance the strength of materials. These ceramic particles are therefore called reinforcement phases. In aluminum alloys, adding copper, silicon, magnesium, and other metal elements to the aluminum matrix to form a solid solution and precipitate phase can enhance the strength of aluminum alloys through heat treatment [1]. However, aluminum alloys have lower hardness and strength than other alloys, and their significant thermal expansion coefficient also limits their application under high-temperature conditions. To address these issues, ceramic particles were added to improve their hardness, strength, high-temperature stability, and low thermal expansion coefficient [2,3]. Aluminum matrix composite (AMC) materials generally refer to composite materials made from an aluminum alloy as the substrate. With the deepening of research on aluminum-based composite materials, the research quantity and application scope of these materials are increasing year by year, and they have been widely used in many fields such as automobiles [4], aerospace [5], electronic packaging [6], and so on.

The commonly used aluminum-based silicon carbide composite material, although it has been applied in various fields, still has some problems that need to be solved. Severe pitting corrosion will occur in environments containing chloride ions [7,8]. Compared to aluminum alloys, this galvanic corrosion will accelerate the breakdown of the material [9]. Despite the existence of corrosion issues, research on the corrosion behavior and corrosion protection of aluminum matrix composites currently needs to be improved. Traditionally, anodizing is a cost-effective method for improving the corrosion resistance of aluminum alloys, resulting in the production of anodized aluminum oxide films (AAO) [10,11]. However, studies have shown that the presence of silicon carbide particles has a negative impact on the anodic oxidation treatment of aluminum matrix composites. The addition of 1 wt% and 5 wt% nano-SiC particles reduces the efficiency of the anodic oxidation process, resulting in an uneven thickness of the oxide layer. And when the additional amount reaches 10 wt%, the particles will hinder the formation of a continuous oxide film [12].

Among many surface modification technologies, atomic layer deposition (ALD), with its unique layer-by-layer film-forming method, can prepare superior anti-corrosion oxide films and provide a feasible solution [13]. Atomic layer deposition, which has attracted wide attention recently, creates a flat-coated film with low defect density and a non-porous structure. Because the forming chemical reaction occurs on the material surface, ALD offers excellent consistency and extremely high step coverage, making it suitable for film deposition with a high aspect ratio [14,15]. In this study, we fabricated various ALD-deposited oxide films on aluminum matrix composites, identified the oxide film offering the most effective corrosion protection for aluminum-based composites, and investigated its corrosion protection mechanism.

## 2. Experimental Methods

### 2.1. Sample Preparation

The AMC used in this study was provided by Huang Chieh Metal Composite Material Tech. Co., Ltd., New Taipei, Taiwan, was produced via hot isostatic pressing (HIP) with 6061 aluminum alloy powder added with 20 volume percent of silicon carbide particles, and the average particle size of silicon carbide was 0.7 μm. After sintering, the AMC was extruded and rolled to form a 1.1 mm thick plate. This plate was subsequently cut into test pieces of various sizes based on the experimental requirements.

Before the ALD process, all test pieces were ground and polished to remove surface defects originating from the natural oxide layer and prior processing steps. The grinding began with SiC sandpapers from coarse to fine, followed by 800 #, 1000 #, 1500 #, and 2500 # sandpapers, and then polished with 3 μm and 1 μm diamond solutions to ensure perfectly flat surfaces. After grinding and polishing, the test pieces were soaked in acetone and placed in an ultrasonic cleaner for 20 min. Finally, the pieces were rinsed with deionized water and dried with a high-purity nitrogen gun, maintaining surface cleanliness.

### 2.2. Atomic Layer Deposition

The plasma-enhanced ALD (PE-ALD) system model (Fiji model, manufactured by Cambridge NanoTech Inc., located in Waltham, MA, USA) was employed to deposit high-quality oxide films on the AMC surface. The deposition took place at a temperature of 250 °C and a pressure of 3 × 10^−1^ Torr. Each processing cycle consisted of four steps: a 30 mTorr precursor pulse, a 15 s argon purge, a 20 s 300 W oxygen plasma treatment, and another 20 s argon purge. The precursors used for the four oxide films—Al_2_O_3_, ZrO_2_, TiO_2_, and HfO_2_—were Trimethylaluminum (TMA, >99% Strem Chemicals, Newburyport, MA, USA), Tetrakis(dimethylamino) zirconium (TDMAZr, >99% Strem Chemicals, Newburyport, MA, USA), Tetrakis(dimethylamino) titanium (TDMATi, >99% Strem Chemicals, Newburyport, MA, USA), and Tetrakis(dimethylamino) hafnium (TDMAHf, >99% Strem Chemicals, Newburyport, MA, USA), respectively. The respective precursor doses were 0.01 s, 0.7 s, 0.8 s, and 0.7 s. The oxygen plasma consisted of a gas mixture with 20 sccm of oxygen and 200 sccm of argon. According to different research purposes, this study was divided into two parts:

In the first part, Al_2_O_3_, ZrO_2_, TiO_2_, and HfO_2_ oxide films were each deposited on the AMC surface for 200 cycles. This part compared the corrosion protection offered with each oxide film to the AMC.

The second part exclusively focused on depositing hafnium dioxide on the specimens for 50, 100, 150, and 200 cycles, aiming to study the influence of different oxide film thicknesses on enhancing corrosion resistance.

### 2.3. Characterization and Analysis

The surface morphology of the bare specimen was observed using the secondary electron image of the electron probe microanalyzer (EPMA) (JEOL JXA-8530F plus FE-EPMA, Tokyo, Japan), and the element distribution of the substrate was analyzed through surface scanning.

After the ALD process, the degree of crystallinity and the crystal structure of the deposited films were examined using a high-power (15 kW) Diffractometer (Rigaku TTRAX 3, Tokyo, Japan). Grazing incidence X-ray diffraction (GIXRD) was performed to analyze the crystallinity and crystal structure of the oxidized films. For this procedure, the incident beam was maintained at a small angle (ω) less than 3°, while the detector operated from 20° to 80° at 2θ. The step value was set at 0.04° with a 4°/min scanning rate.

The film thickness was measured, and its crystallinity was verified using a transmission electron microscope (TEM) (FEI Tecnai G2 F20, FEI Company, Hillsboro, OR, USA). TEM specimens were prepared using a dual-beam FIB and electron beam system (FEI Helios 600i, FEI Company, Hillsboro, OR, USA). Platinum and carbon layers were deposited onto the surface of AMC specimens as protective and conductive layers and subsequently cut and thinned to approximately 100 nm using a gallium ion beam. A TEM equipped with an energy-dispersive X-ray spectrometer (EDS) (Genesis, EDAX Japan, Tokyo, Japan) was utilized to capture images at 200 kV. Additionally, EDS element mapping was performed to confirm element distribution and identify the composition of thin films prepared on AMC. High-resolution TEM (HRTEM) images were captured in the bright field, and the crystallization of the thin film was analyzed using a fast Fourier transform.

Finally, Al Kα X-ray photoelectron spectroscopy (XPS) (Thermo Scientific K-Alpha, Thermofisher Scientific, E. Grinstead, UK) with a radiation source (E = 1.486 keV) was employed to measure the valence states of the ALD thin films. Three points were analyzed for each sample to determine the composition of the thin films.

### 2.4. Corrosion Evaluation

#### 2.4.1. Electrochemical Test Overview

The corrosion performance of various specimens was evaluated using a potentiostat (VersaSTAT 3, AMETEK, Berwyn, PA, USA) at room temperature. All experiments employed a three-electrode electrochemical cell consisting of a working electrode (WE), a reference electrode (RE), and a counter electrode (CE). The WE was an AMC specimen with a reactive area of 1.767 cm^2^, the RE was a saturated calomel electrode, and the CE was a platinum sheet. The entire experimental setup was housed within a Faraday cage. A 1.5 wt% NaCl solution served as the test electrolyte. Before each measurement, the open circuit potential (OCP) was performed to reach a steady state. If potential variations remained below 20 mV for 500 s, the system was deemed stable, prompting the commencement of measurements.

#### 2.4.2. Electrochemical Impedance Spectroscopy (EIS)

To explore the corrosion resistance of AMC in a chloride-rich environment, EIS tests were conducted. Data spanning a frequency range of 10^5^ to 10^−2^ Hz were collected, and the impedance value and phase angle under A.C. voltage feedback were recorded. The data were plotted on a Nyquist diagram and fitted with an equivalent circuit using ZSimpWin software (version 3.6) to simulate the equivalent circuit model of the impedance spectrum.

#### 2.4.3. Potentiodynamic Polarization Measurements (PDP)

Potentiodynamic polarization measurements were initiated from the OCP and recorded the current values at a scan rate of 0.5 mV/s within a range of −0.4 V to +2.3 V. The potential with the least current density is termed the corrosion potential (E_corr_). Data within the potential’s linear range of ±120 mV were processed using the Tafel extrapolation method to derive the corrosion current density (I_corr_). Each specimen’s I_corr_ served as a quantitative measure of its corrosion rate [16].

#### 2.4.4. Salt Spray Test

According to the ASTM B1117-03 standard [17], a salt spray test was conducted using a 5 wt% NaCl solution at 47 °C and a pressure of 1 kg/cm². Before testing, specimens were prepared by shielding their peripheries with corrosion-resistant tape, exposing only a central area of a 20 mm × 20 mm square to the salt spray. The entire test duration was 216 h, during which the condition of corrosion was periodically recorded. Based on the ISO 10289:2001 guidelines [18], the proportion of the corroded area to the entire area determined the sample’s rating. A pristine specimen with no defects or changes on the surface received a rating of 10. Corrosion areas covering up to 0.1%, 0.1–0.25%, 0.25–0.5%, 0.5–1.0%, 1.0–2.5%, 2.5–5%, 5–10%, 10–25%, and 25–50% of the total area received ratings of 9, 8, 7, 6, 5, 4, 3, 2, and 1, respectively, with 1 indicating severe corrosion.

#### 2.4.5. Immersion Test

To evaluate the potential corrosion behavior, specimens were immersed in a 1.5 wt% NaCl solution for 4 or 8 h. After corrosion, scanning electron microscopy (SEM) was applied to observe the changes in surface morphology. Additionally, EPMA at a 15 kV accelerating voltage analyzed the elemental distribution on the specimen surfaces after corrosion. Before EPMA, all specimens were coated with a carbon layer to minimize charge accumulation during the analysis.

## 3. Results and Discussion

### 3.1. Characterization of AMC

**Figure 1:**
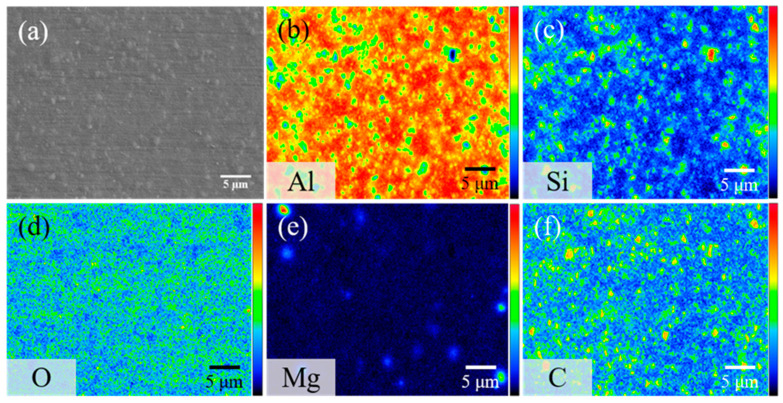
(**a**) Secondary electron image of AMC and its corresponding elemental mappings showing the distribution of (**b**) Al, (**c**) Si, (**d**) O, (**e**) Mg, and (**f**) C.

**Figure 2:**
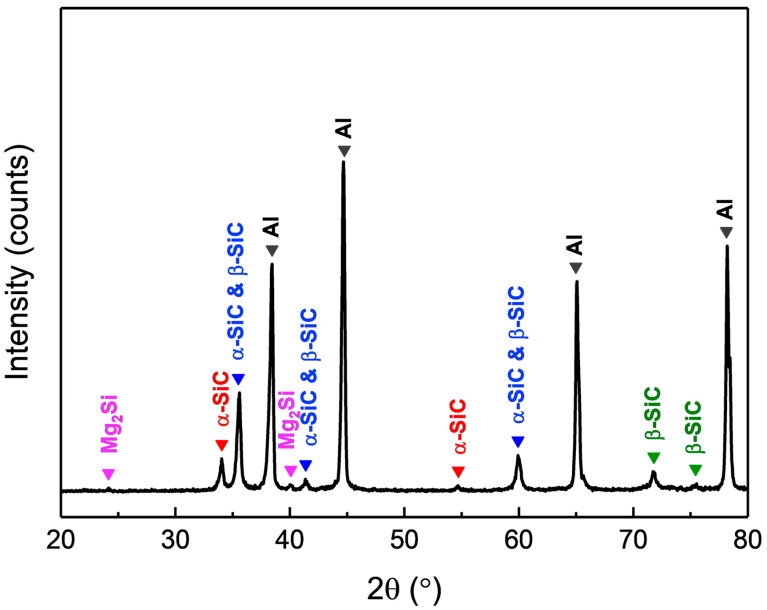
XRD pattern of AMC.

The surface morphology of AMC and the distribution of SiC particles within the aluminum alloy substrate are characterized using EPMA, specifically through secondary electron imaging and elemental surface scanning. Figure 1a presents a secondary electron image showing the surface morphology of the AMC specimen, which reveals that particles are randomly embedded in the matrix. The elemental mapping shown in Figure 1b–f indicates that the particles consist primarily of SiC, while the matrix is predominantly the aluminum alloy. Notably, the SiC particles are approximately 1 μm in size and are evenly distributed within the aluminum alloy matrix. The detected Mg signal likely arises from Mg_2_Si precipitates present in the 6061 aluminum alloy [19]. Figure 2 shows the X-ray diffraction pattern of AMC, with dominant peaks corresponding to crystalline aluminum. Two discernible SiC phases are present: α-SiC, characterized by a wurtzite-like crystalline structure, and cubic β-SiC [20]. Additionally, minor peaks associated with Mg_2_Si suggest a trace amount of this phase. By consolidating insights from SEM, elemental mapping, and XRD, we can conclude that the AMC composite primarily consists of an Al matrix with interspersed SiC particles and contains traces of Mg_2_Si.

### 3.2. Atomic Layer Deposition

#### Film Characteristics

**Figure 3:**
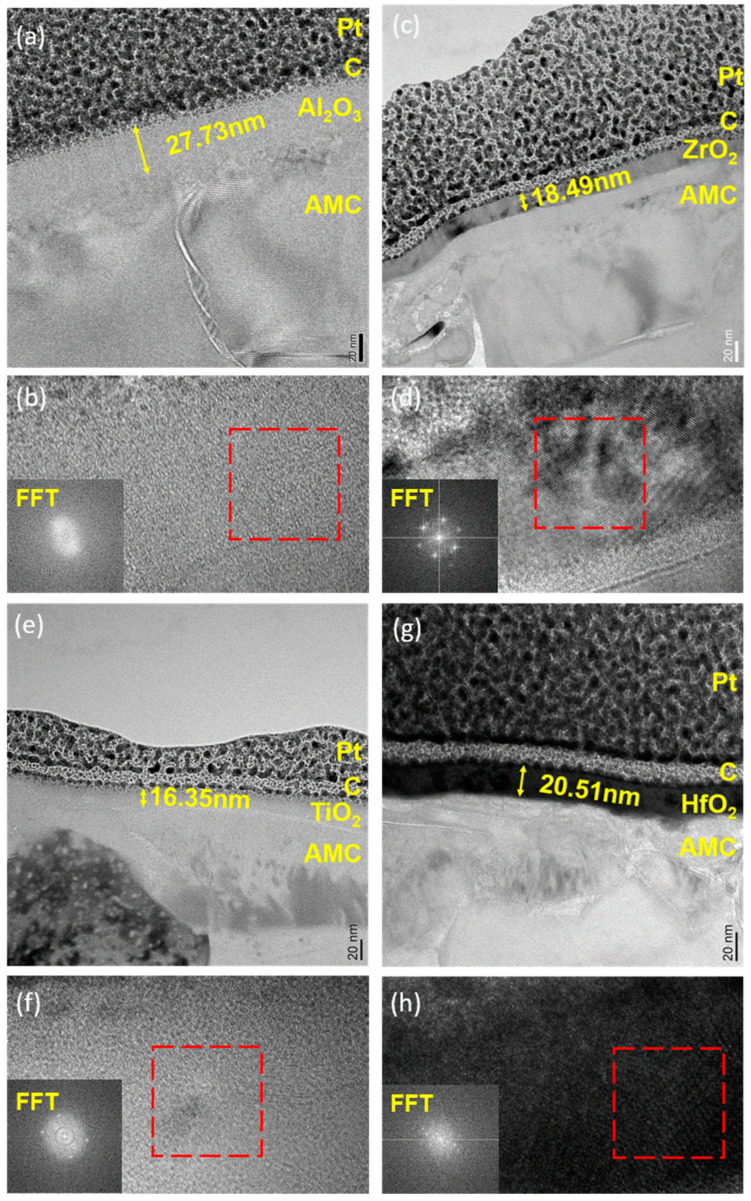
(**a**) TEM bright field image, (**b**) HRTEM image and FFT patterns of Al_2_O_3_/AMC; (**c**) TEM bright field image, (**d**) HRTEM image and FFT patterns of ZrO_2_/AMC; (**e**) TEM bright field image, (**f**) HRTEM image and FFT patterns of TiO_2_/AMC; (**g**) TEM bright field image, (**h**) HRTEM image and FFT patterns of HfO_2_/AMC. The regions encircled in red boxes in subfigures b, d, f, and h indicate the areas where Fast Fourier Transform (FFT) analysis was performed.

Figure 3 depicts cross-sectional images of AMC coated with various oxide layers. Despite the heterogeneous structure of the Al alloy and SiC particles on the AMC surface, the deposited film adheres uniformly to the entire substrate. This uniformity can be attributed to the surface reactions and self-limiting nature of ALD [14]. Figure 3a presents a TEM cross-sectional image of Al_2_O_3_ deposited on AMC (Al_2_O_3_/AMC). The ALD film covers the substrate uniformly, with a thickness of approximately 27.73 nm. However, the ALD-grown Al_2_O_3_ layer is indistinguishable from the naturally oxidized Al_2_O_3_ on the Al substrate. Figure 3b shows the HRTEM image of Al_2_O_3_/AMC. The inset displays the FFT pattern from the designated area inside the red frame, without interference fringes or diffraction spots, indicating the amorphous structure of the Al_2_O_3_ film. Figure 3c displays the TEM cross-sectional image of AMC coated with a ZrO_2_ film (ZrO_2_/AMC). The thickness of the film is measured to be 18.49 nm, and the growth rate per cycle is 0.09245 nm. In Figure 3d, the HRTEM image reveals the crystalline nature of the ZrO_2_ film deposited on AMC. This is evidenced with the visible diffraction spots in the inserted FFT pattern. Figure 3e presents a cross-sectional view of TiO_2_ deposited on AMC (TiO_2_/AMC). The TiO_2_ film with a thickness of 16.35 nm has a growth rate of 0.08175 nm per cycle. Figure 3f shows the HRTEM image of TiO_2_/AMC. The inserted FFT pattern displays diffraction spots and rings, indicating that the TiO_2_ film has mixed amorphous and crystalline structures. Figure 3g shows that the HfO_2_ film deposited on AMC has a thickness of 21.33 nm and a growth rate of 0.1067 nm/cycle. An additional layer between the oxide and AMC is noted, likely native oxide on the Al alloy [21]. Lastly, Figure 3h presents the HRTEM image of HfO_2_/AMC. Interference fringes in the highlighted area and the diffraction spots and rings in the inset indicate that the HfO_2_ film is a mixture of amorphous and crystalline structures.

**Figure 4:**
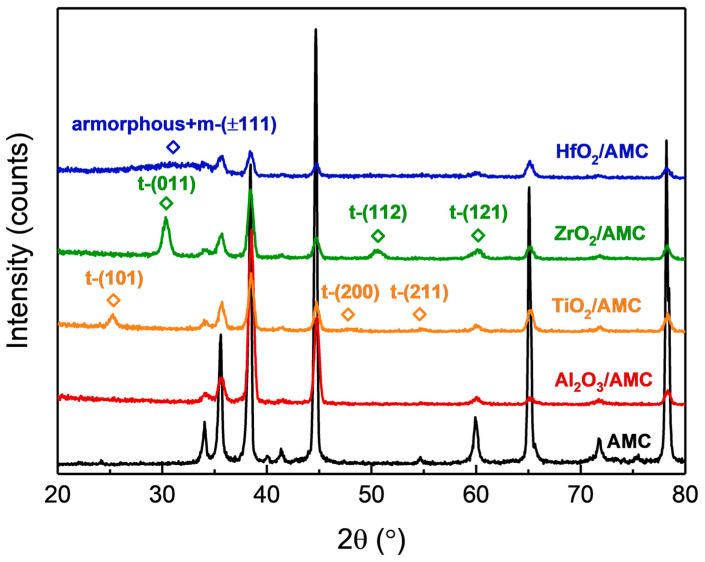
GIXRD patterns of Al_2_O_3_/AMC, ZrO_2_/AMC, TiO_2_/AMC, and HfO_2_/AMC.

The crystalline state of the films observed with TEM can be verified using the XRD results. Figure 4 displays the GIXRD patterns of various specimens. For the Al_2_O_3_/AMC pattern, no peaks other than those of AMC are detected. In conjunction with the HRTEM image in Figure 3b, it can be concluded that the Al_2_O_3_ film is amorphous. The GIXRD pattern of ZrO_2_/AMC reveals three distinct peaks, which align with the diffraction signals of (011), (112), and (121) planes of tetragonal ZrO_2_ [22]. Combined with the HRTEM image in Figure 3d, it is deduced that the ZrO_2_ film fabricated on AMC is polycrystalline and belongs to the tetragonal system. The GIXRD pattern of TiO_2_/AMC exhibits three peaks correlating with the diffraction signals of (101), (200), and (211) planes of tetragonal TiO_2_ in the anatase phase [23]. In combination with the HRTEM image in Figure 3f, it is posited that the TiO_2_ film formed on AMC is a polycrystalline anatase phase. Notably, in the GIXRD pattern of HfO_2_/AMC, there are no prominent peaks for HfO_2_, but a broad peak is observed between 25° and 35°. Paired with the HRTEM image in Figure 3h and the FFT pattern, it is confirmed that the HfO_2_ film deposited on AMC at 250 °C comprises both amorphous and crystalline monoclinic structures. A previous study [24] reported that HfO_2_ films deposited at lower temperatures had similar XRD patterns. Given that the deposition temperature in this study was 250 °C, some HfO_2_ may not achieve a crystalline phase, resulting in the coexistence of crystalline and amorphous structures as discerned with both XRD and TEM.

**Figure 5:**
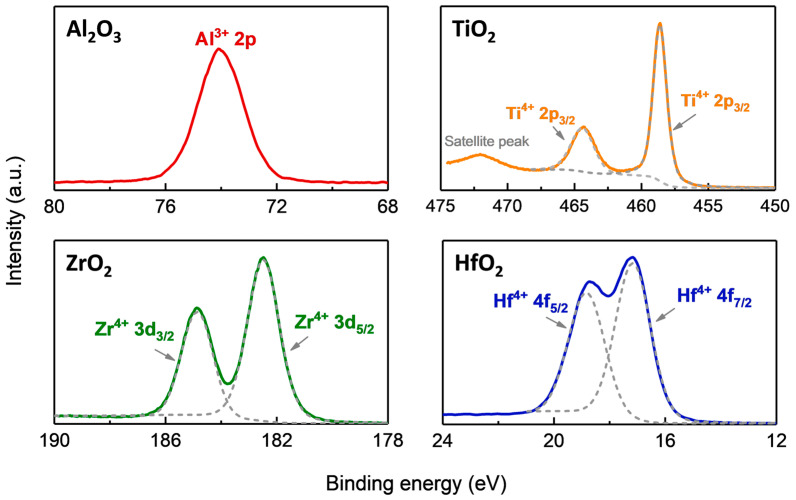
High-resolution XPS scans of Al_2_O_3_, ZrO_2_, TiO_2_, and HfO_2_ films.

X-ray photoelectron spectroscopy is used to analyze the composition of the four oxide films. The analyzed results are shown in Figure 5 Compared to the other three oxide films, in Figure 5, only a characteristic peak of Al_2_O_3_ is observed at 74.1 eV, corresponding to Al^3+^ 2p in Al_2_O_3_ [25]. This indicates that the composition of alumina is Al_2_O_3_. Similarly, in Figure 5, the two peaks of ZrO_2_ are located at 185.3 eV and 182.9 eV, corresponding to the characteristic binding energies of Zr^4+^ 3d_3/2_ and Zr^4+^ 3d_5/2_ in ZrO_2_ [26]. We infer that the ZrO_2_ film grown on AMC has a composition ratio of 1:2. In Figure 5, for TiO_2_, in addition to the characteristic peaks of Ti^4+^ 2p_1/2_ and Ti^4+^ 2p_3/2_ located at 464.6 eV and 458.9 eV, respectively, satellite peaks corresponding to TiO_2_ are also observed [27], indicating that the composition of the deposited film is TiO_2_. For HfO_2_, the peaks of 18.82 eV and 17.16 eV in the XPS spectrum are characteristic peaks of Hf^4+^ 4f_5/2_ and Hf^4+^ 4f_7/2_ in HfO_2_, respectively [28].

### 3.3. Corrosion Characteristics

#### 3.3.1. Electrochemical Analysis

**Figure 6:**
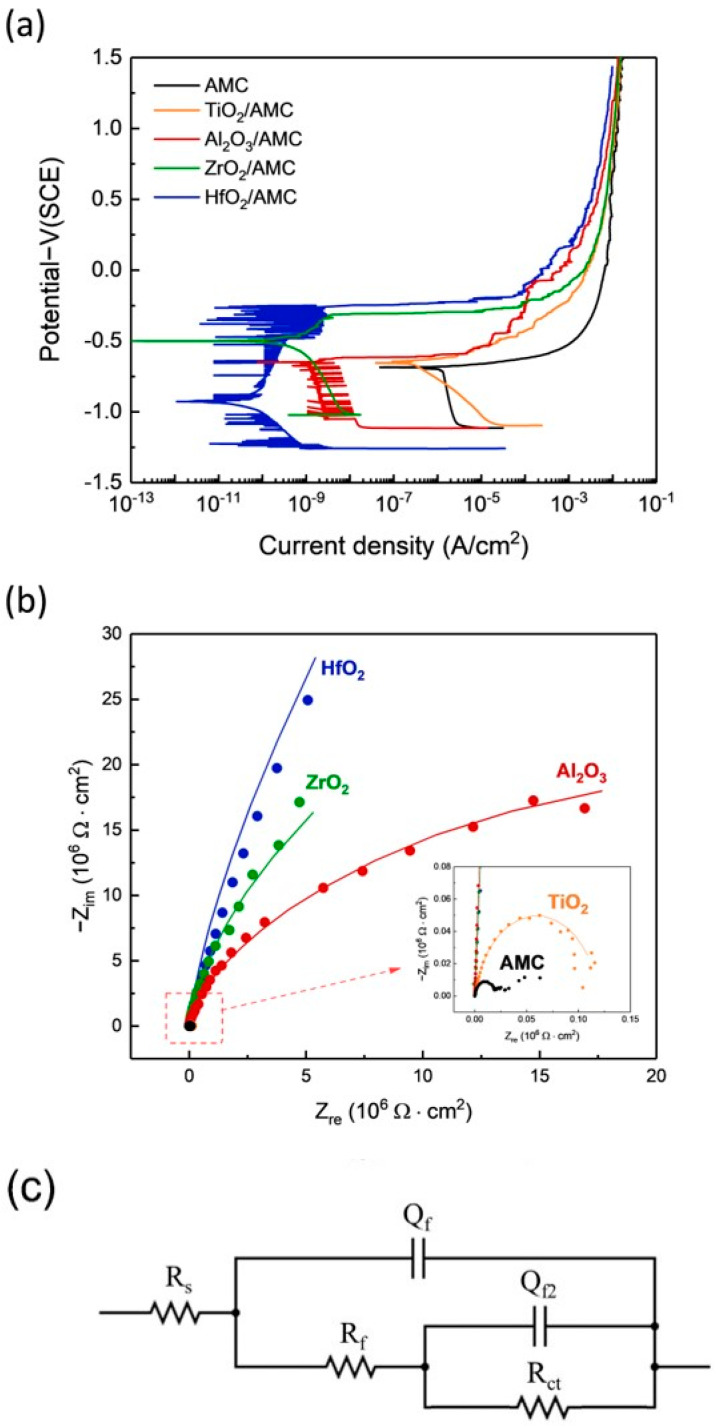
(**a**) PDP curves, (**b**) Nyquist plots of AMC coated with Al_2_O_3_, ZrO_2_, TiO_2_, and HfO_2_ films, (**c**) equivalent circuit model used for interpreting the results of the Nyquist plot.

The electrochemical corrosion rates are determined using potentiodynamic polarization (PDP) curves. The current density at zero overpotential is termed “corrosion current density”, typically denoted as i_corr_, and is directly correlated with the corrosion rate of the specimen [29]. Figure 6a shows the polarization curves for bare AMC and AMC coated with Al_2_O_3_, ZrO_2_, TiO_2_, and HfO_2_ films. Their corresponding values are enumerated in Table 1. The data reveal that AMCs coated with Al_2_O_3_, ZrO_2_, TiO_2_, and HfO_2_ films all display a reduction in current density, signifying that the application of ALD films successfully diminishes the corrosion rate of AMC. Notably, the four distinct oxide films provide different levels of corrosion protection. The TiO_2_/AMC has an i_corr_ value of 4.66 × 10^−7^ A/cm^2^, reducing i_corr_ by less than an order of magnitude. The Al_2_O_3_/AMC exhibits an i_corr_ of 2.56 × 10^−9^ A/cm^2^, and the ZrO_2_/AMC has an i_corr_ of 9.28 × 10^−10^ A/cm^2^. Both reduce AMC’s i_corr_ by three and four orders of magnitude, respectively. Among them, the HfO_2_/AMC showcases the lowest value, at 5.89 × 10^−11^ A/cm^2^, signifying that the HfO_2_ film drastically reduces AMC’s i_corr_ by a significant five orders of magnitude.

Figure 6b shows the Nyquist plots of AMC coated with Al_2_O_3_, ZrO_2_, TiO_2_, and HfO_2_ films and bare AMC. It shows that the diameters of Al_2_O_3_/AMC, ZrO_2_/AMC, and HfO_2_/AMC are much larger than those of bare AMC, and the curve of bare AMC is almost invisible in Figure 6b, indicating that the Al_2_O_3_, ZrO_2_, and HfO_2_ films provide good corrosion protection for AMC in the NaCl_(aq)_ solution. The inset in Figure 6b is a partially enlarged view of the red square area, showing that the TiO_2_/AMC has a larger diameter than the bare AMC, indicating that the TiO_2_ film can provide a certain degree of corrosion protection, but the protective effect is not significant, which is consistent with the results of PDP. The fitting curves in Figure 6b are obtained by fitting with the equivalent circuit model shown in Figure 6c, and the fitted circuit components and parameters are shown in Table 2: Q_f_ and Q_f2_ are the constant phase components provided with the ALD film and naturally formed Al_2_O_3_ film, respectively, R_s_ is solution impedance, R_f_ is mass transfer impedance provided with the ALD film, and R_ct_ is charge transfer impedance caused by pitting corrosion. The impedance of the equivalent circuit can be written as Formula (1).

(1) $$Z={R_{s}+\left[\frac{1}{{\left(\frac{1}{R_{ct}}+({j\omega )}^{n}C_{f2}\right)}^{-1}+R_{f}}+{\left(j\omega \right)}^{n}C_{f}\right]}^{-1}$$

The polarization impedance (R_p_), as defined in Table 2, is the sum of R_f_ and R_ct_. It is used to evaluate the corrosion resistance of the material system [30]. The R_p_ values rank in the following descending order: HfO_2_, ZrO_2_, Al_2_O_3_, and TiO_2_. The most significant decrease in corrosion current density observed in HfO_2_/AMC is attributed to the high polarization impedance of HfO_2_.

**Table 2:** Fitted circuit components and parameters of AMC coated with Al_2_O_3_, ZrO_2_, TiO_2_, and HfO_2_ films.

	R_s_ (Ω·cm^2^)	Q_f_ (S·s^−n^·cm^2^)	n_f_	R_f_ (Ω·cm^2^)	Q_f2_ (S·s^−n^·cm^2^)	n_f2_	R_ct_ (Ω·cm^2^)	R_p_ (Ω·cm^2^)
**Al_2_O_3_**	115.7	2.60 × 10^−7^	0.97	7.81 × 10^6^	1.10 × 10^−7^	0.57	5.22 × 10^7^	1.97 × 10^7^
**ZrO_2_**	126.7	3.94 × 10^−7^	0.99	2.28 × 10^2^	3.34 × 10^−7^	0.87	9.13 × 10^7^	9.19 × 10^7^
**TiO_2_**	126.1	1.21 × 10^−6^	0.92	2.37 × 10^2^	1.13 × 10^−5^	0.84	1.20 × 10^5^	1.20 × 10^5^
**HfO_2_**	156.4	4.56 × 10^−7^	0.96	1.53 × 10^7^	7.05 × 10^−8^	0.74	7.29 × 10^8^	5.22 × 10^8^

**Figure 7:**
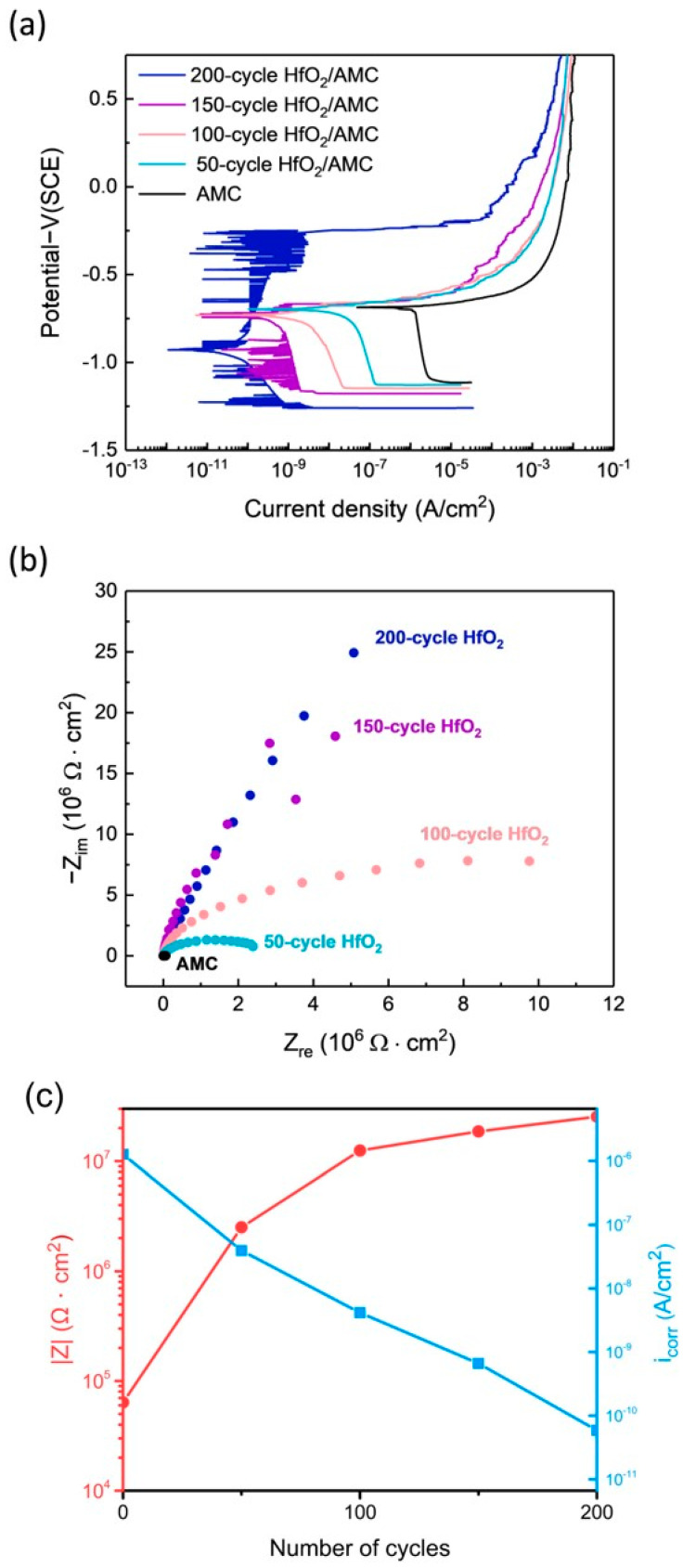
(**a**) PDP curves, (**b**) Nyquist plots of AMC coated with HfO_2_ films of different thicknesses, (**c**) impedance at 0.01 Hz as a function of HfO_2_ film thickness and corresponding current density.

Given that AMC coated with the HfO_2_ film exhibits the lowest corrosion rate among the four oxide films, to further investigate the effect of HfO_2_ film thickness on the corrosion protection of AMC, the polarization curves of AMC coated with HfO_2_ for 200 cycles, 150 cycles, 100 cycles, and 50 cycles, as well as bare AMC, are tested. The experimental results are illustrated in Figure 7a, and their corresponding E_corr_ and i_corr_ values are tabulated in Table 3. Compared with the bare AMC, all four specimens coated with HfO_2_ films of different thicknesses exhibit lower i_corr_ values, and the i_corr_ value decreases with the increasing deposition cycle of the HfO_2_ film. Specifically, the i_corr_ values of AMC coated with HfO_2_ films for 200, 150, 100, and 50 cycles are 5.89 × 10^−11^ A/cm^2^, 6.63 × 10^−10^ A/cm^2^, 4.15 × 10^−9^ A/cm^2^, and 3.94 × 10^−8^ A/cm^2^, respectively. In comparison, the i_corr_ for the bare AMC is 1.27 × 10^−6^ A/cm^2^. This indicates that the corrosion rate decreases with the increasing deposition cycle of the HfO_2_ film.

Figure 7b also depicts the Nyquist plots of AMC coated with HfO_2_ films of different thicknesses. It shows that even for the HfO_2_ film deposited with only 50 cycles, the diameter of the Nyquist plot is much larger than that of the bare AMC. This suggests that a thin HfO_2_ film can substantially improve the corrosion impedance of AMC. As the deposition cycle of the HfO_2_ film increases, the diameter of the Nyquist plot increases, indicating that the corrosion impedance increases with the increase in film thickness. This observation is reinforced with Figure 7c, which charts the impedance variance with film thickness against the corresponding corrosion current density at a frequency of 0.01 Hz. The findings further demonstrate that impedance escalates with film thickness, correlating with a decrease in corrosion current density as the film thickens.

#### 3.3.2. Salt Spray Test

**Figure 8:**
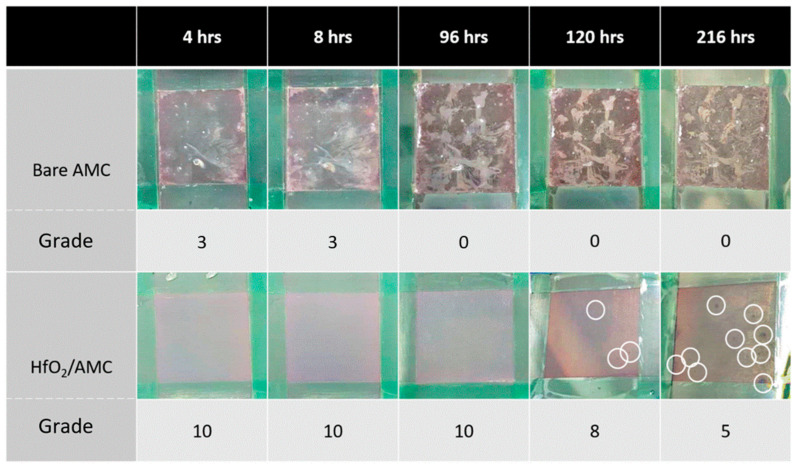
Results of salt spray tests on HfO_2_/AMC and bare AMC.

The results of the salt spray test are shown in Figure 8. Ratings are assigned based on the specified standard [18]. After 4 h of salt spray exposure, the bare AMC starts showing signs of corrosion pitting in multiple areas, receiving a rating of three. By 96 h, the extent and size of these corrosion pits increase considerably, resulting in a downgrade in its rating to 0. Beyond 120 h, the surface corrosion intensifies—not so much in the area but in depth.

Conversely, the HfO_2_/AMC surface remains corrosion-free for up to 96 h, maintaining an impeccable rating of 10. However, corrosion signs begin emerging on the HfO_2_/AMC surface after 120 h, as indicated with the white circles in Figure 8, bringing its rating down to 8. Despite this, the corrosion on HfO_2_/AMC specimens is markedly less severe than on the bare AMC. The prolonged protection provided with the HfO_2_ coating underscores its effectiveness in safeguarding AMC against corrosion even in adverse conditions. By 216 h, significant corrosion pits appeared on the HfO_2_/AMC, resulting in a further rating drop to 5. In summary, the results of the salt spray test demonstrate the protective prowess of the HfO_2_ coating in enhancing AMC’s corrosion resistance. While the bare AMC showed signs of degradation as early as 4 h into the test, the HfO_2_ coating effectively prolonged its durability. This evidence suggests that HfO_2_ coating, applied via ALD, holds promising potential for wide-ranging applications in industry.

#### 3.3.3. Corrosion Behaviors

**Figure 9:**
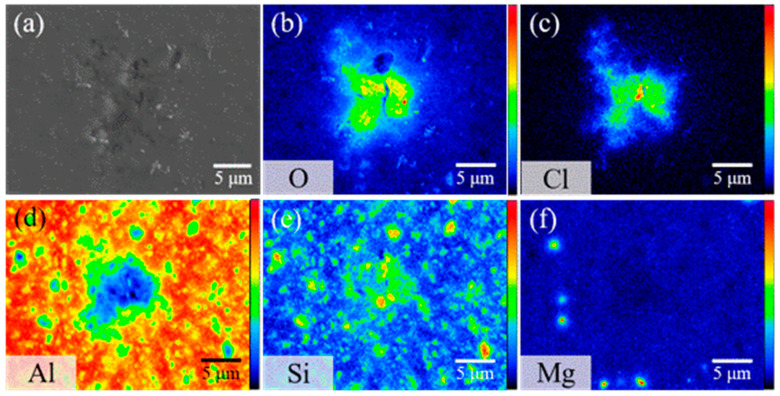
(**a**) Secondary electron image and (**b**–**f**) elemental mappings of bare AMC after 8 h of immersion in 1.5% NaCl_(aq)_ solution, showing the distribution of (**b**) O, (**c**) Cl, (**d**) Al, (**e**) Si, and (**f**) Mg.

After immersing the bare AMC in a NaCl (aqueous) solution for 8 h, EPMA was used to perform an element mapping to infer chemical corrosion reactions. The analysis reveals corrosion pits at multiple locations and provides insight into the composition of the corrosion products and the corrosion behaviors of AMC. Figure 9 illustrates the elemental distribution map of AMC after 8 h of immersion. Scans focus on early-stage corrosion points to discern the effects of Mg and Si particles on AMC. The results show a higher concentration of SiC particles at corrosion points than in surrounding regions. In contrast, areas free from corrosion predominantly feature the aluminum alloy substrate with minimal SiC content. This suggests that micro-cracks between SiC particles and the Al matrix may be the focal points for initiating corrosion. Notably, due to these micro-cracks, corrosion appears to preferentially initiate in regions rich in SiC particles [31]. The influence of the intrinsic precipitate, Mg_2_Si, is not accounted for, given its negligible content.

**Figure 10:**
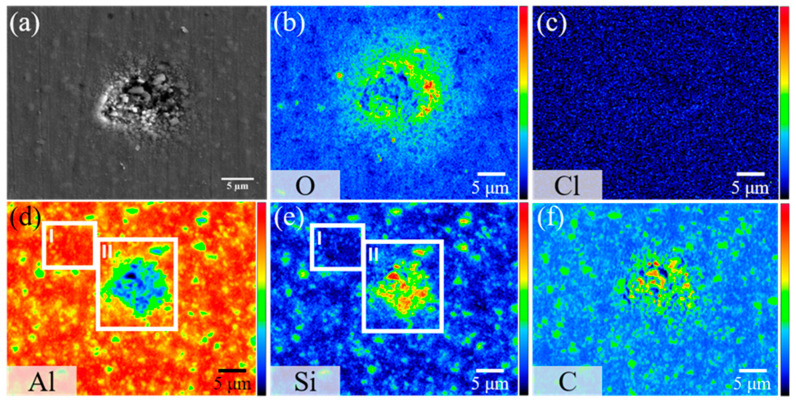
(**a**) Secondary electron image and (**b**–**f**) elemental mappings of a corrosion pit on bare AMC after 8 h of immersion in 1.5% NaCl_(aq)_ solution, showing the distribution of (**b**) O, (**c**) Cl, (**d**) Al, (**e**) Si, and (**f**) C.

As presented in Figure 10, further exploration of elemental distribution after 8 h of immersion unveils distinct O and Cl^−^ signals surrounding the corrosion sites. This implies that the corrosion byproducts comprise mainly aluminum oxides and chlorides. In the presence of chlorides, aluminum tends to produce aluminum hydroxide, Al(OH)_3_, simultaneously releasing hydrogen ions, H^+^. Under these acidic conditions, aluminum hydroxide may further react with sodium chloride, yielding aluminum chloride, AlCl_3_, and water [32].

Two marked regions, Points I and II, on the Al and Si elemental distribution maps (Figure 10) provide additional clarity. Point II reveals a dense SiC particle concentration, whereas Point I has barely any SiC presence. A comparison between these two points highlights a difference in corrosion patterns within the Al matrix and SiC-rich regions. Point II suffers from intense pitting corrosion, whereas Point I remains intact. This observation confirms that AMC corrosion in NaCl predominantly targets SiC-rich areas. After immersion, the Al signal at Point II diminishes considerably, yet some SiC particles are still discernible. This suggests that the micro-cracks between Al and SiC can serve as primary points for corrosion onset, altering the material’s resistance to corrosion. When such corrosion is initiated at these micro-cracks, the pits can expand, coalescing into extensive corrosion zones, which may culminate in structural failures. This leads to a corroded aluminum alloy substrate, leaving SiC particles exposed [33].

**Figure 11:**
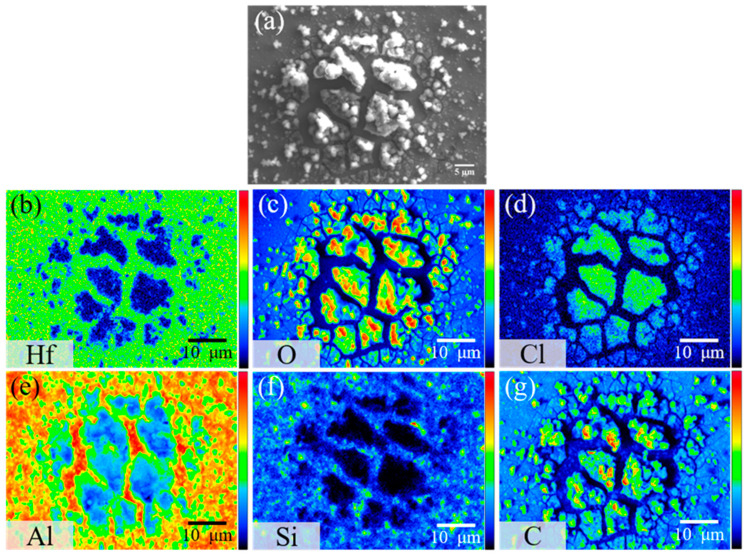
(**a**) Secondary electron image and (**b**–**g**) elemental mappings of HfO_2_/AMC after 8 h of immersion in 1.5% NaCl_(aq)_ solution, showing the distribution of (**b**) Hf, (**c**) O, (**d**) Cl, (**e**) Al, (**f**) Si, and (**g**) C.

After 8 h of immersion, the AMC coated with 200 cycles of the HfO_2_ film exhibits a distinct surface morphology, as depicted in Figure 11a. Rather than pits, cracks appear on the specimen surface. This can be attributed to the ALD-HfO_2_ coating acting as a diffusion barrier, limiting direct interaction between the corrosive environment and the underlying aluminum and ceramics. Consequently, fewer corrosion ions penetrate the oxide layer, changing the corrosion behavior from wide-scale pitting to localized exfoliation. Additionally, corrosion does not progress along the Al-SiC interface to significant depths. Element mapping diagrams in Figure 11b–f suggest that the corrosion products, comprising oxides and chlorides, are similar to those on bare AMC. These products accumulate between the cracks. The protective ability of the HfO_2_ film is evident, as it mitigates micro-crack-induced corrosion and postpones pitting corrosion. The absence of Hf and Si signals within the corrosion products suggests that the underlying Al corrodes, leading to the detachment of the overlying HfO_2_ film and associated SiC particles. However, the presence of Hf at the cracks signifies that the HfO_2_ film, even after corrosion, offers some protective effect.

**Figure 12:**
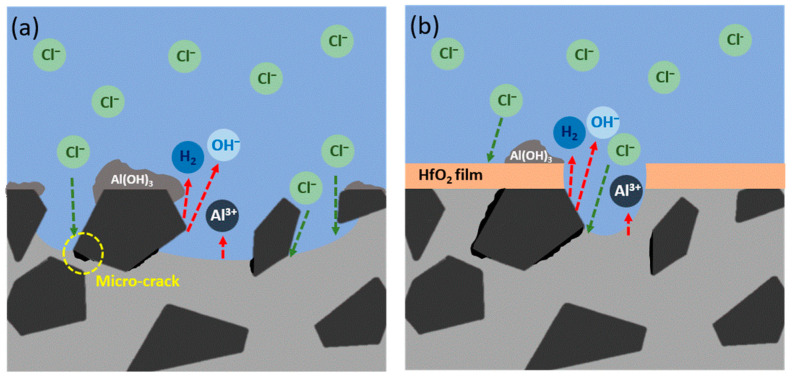
Schematic diagrams of corrosion behaviors in NaCl solution for (**a**) bare AMC and (**b**) ALD-HfO_2_/AMC.

According to the results of EPMA element mapping and corroborated with reference [34], Figure 12a,b graphically represent the corrosion behaviors of both bare and ALD-HfO_2_/AMC in the NaCl_(aq)_ solution. For bare AMCs, the significant influence of SiC particles on corrosion is evident, particularly in forming micro-cracks and directing corrosion. Yet, when AMC is furnished with the ALD-HfO_2_ thin film, it effectively prevents direct alloy-corrosive medium contact, thus altering the corrosion mechanism and outcome from pitting to exfoliation.

## 4. Conclusions

Our study shows that atomic layer deposition (ALD) can be effectively used to enhance the corrosion resistance of aluminum matrix composites (AMC).

1. Among the various thin films we examined, including Al_2_O_3_, ZrO_2_, TiO_2_, and HfO_2_, each for 200 cycles, the HfO_2_ film exhibited the highest level of corrosion resistance.
2. The HfO_2_ film of just 50 cycles remarkably reduced the corrosion rate of AMC, improving its resistance by more than 100 times. Additional cycles of deposition further elevated the performance. Specifically, 100 cycles led to a 1000 times improvement, 150 cycles to a 10^4^ times improvement, and 200 cycles to a 10^5^ times improvement in the corrosion resistance, compared to bare AMC.
3. After an 8 h immersion in a 1.5% NaCl solution, HfO_2_-coated AMCs showed fewer and shallower pits compared to bare AMC.
4. Despite its effectiveness in reducing surface corrosion, the HfO_2_ coating could not mitigate the internal cracks between SiC particles and the Al matrix, limiting its application to surface protection only.
5. The HfO_2_-coated AMC outperformed the uncoated one in salt spray tests, maintaining a level five evaluation even after 216 h, while the bare AMC failed in less than 4 h.

## Figures and Tables

**Table 1:** Corrosion potential and corrosion current density of AMC coated with Al_2_O_3_, ZrO_2_, TiO_2_, and HfO_2_ films.

	AMC	Al_2_O_3_/AMC	ZrO_2_/AMC	TiO_2_/AMC	HfO_2_/AMC
**E_corr_ (V vs. SCE)**	−0.686	−0.649	−0.500	−0.655	−0.928
**I_corr_ (A/cm^2^)**	1.27 × 10^−6^	2.56 × 10^−9^	9.28 × 10^−10^	4.66 × 10^−7^	5.89 × 10^−11^

**Table 3:** Corrosion potential and corrosion current density of AMC coated with HfO_2_ films of different thicknesses.

	200 Cycles	150 Cycles	100 Cycles	50 Cycles	AMC
**E_corr_ (V vs. SCE)**	−0.928	−0.726	−0.730	−0.696	−0.686
**I_corr_ (A/cm^2^)**	5.89 × 10^−11^	6.63 × 10^−10^	4.15 × 10^−9^	3.94 × 10^−8^	1.27 × 10^−6^

## Data Availability

The authors confirm that the data supporting the findings of this study are available within the article.
